# The Surprising Effect of Phenformin on Cutaneous Darkening and Characterization of Its Underlying Mechanism by a Forward Chemical Genetics Approach

**DOI:** 10.3390/ijms21041451

**Published:** 2020-02-20

**Authors:** Kei Takano, Akira Hachiya, Daiki Murase, Akiko Kawasaki, Hirokazu Uda, Shinya Kasamatsu, Yoshiya Sugai, Yoshito Takahashi, Tadashi Hase, Atsushi Ohuchi, Tamio Suzuki

**Affiliations:** 1 Biological Science Research, Kao Corporation, Odawara 250-0002, Japan; takano.kei@kao.com (K.T.);; 2 Planning and Implementation, Kao Corporation, Haga 321-3497, Japan; 3 Biological Science Research, Kao Corporation, Haga 321-3497, Japan; 4 Department of Plastic Surgery, Jichi Medical University, Shimotsuke 329-0498, Japan; 5 Core Technology Sector, Kao Corporation, Sumida 131-0044, Japan; 6 Department of Dermatology, Yamagata University Faculty of Medicine, Yamagata 990-9585, Japan

**Keywords:** phenformin, chemical genetics, melanin, skin pigmentation, keratinocyte, autophagy, 7-dehydrocholesterol reductase

## Abstract

Melanin in the epidermis is known to ultimately regulate human skin pigmentation. Recently, we exploited a phenotypic-based screening system composed of ex vivo human skin cultures to search for effective materials to regulate skin pigmentation. Since a previous study reported the potent inhibitory effect of metformin on melanogenesis, we evaluated several biguanide compounds. The unexpected effect of phenformin, once used as an oral anti-diabetic drug, on cutaneous darkening motivated us to investigate its underlying mechanism utilizing a chemical genetics approach, and especially to identify alternatives to phenformin because of its risk of severe lactic acidosis. Chemical pull-down assays with phenformin-immobilized beads were performed on lysates of human epidermal keratinocytes, and subsequent mass spectrometry identified 7-dehydrocholesterol reductase (DHCR7). Consistent with this, AY9944, an inhibitor of DHCR7, was found to decrease autophagic melanosome degradation in keratinocytes and to intensely darken skin in ex vivo cultures, suggesting the involvement of cholesterol biosynthesis in the metabolism of melanosomes. Thus, our results validated the combined utilization of the phenotypic screening system and chemical genetics as a new approach to develop promising materials for brightening/lightening and/or tanning technologies.

## 1. Introduction

Human skin is characterized by various colors ranging from almost black to nearly colorless and gradations between those [1]. Skin color is dominantly determined by the amount and kind of melanin produced in melanocytes located in the basal layer of the epidermis and its distribution at the surface of the body [2,3]. Skin color has a considerable impact on the impression and the identification of people within and between human communities. It is certain that many people wish to change (lighten/darken) their skin color. Hence, ingredients that can regulate skin pigmentation have the potential not only for developing skin lightening/tanning products according to the needs of consumers but also for treating cutaneous pigmentary disorders such as hyperpigmentation (e.g., melasma, senile lentigines, freckles and malignant melanoma) and hypopigmentation (e.g., vitiligo) in the medical field [4].

Historically, phenotypic screens, i.e., assays designed to examine the effects of compounds on cells, tissues or whole organisms, have been the mainstay of drug discovery, especially in the exploration of first-in-class therapeutics. The strength of the phenotypic approach lies in that those assays do not require prior understanding of the compounds’ modes of actions. Thus, the results obtained from those assays can be translated into a phase assessing their in vivo efficacy in humans more effectively than target-based approaches [5]. In our previous studies, we established an *ex vivo* skin culture system and applied that system to evaluate the skin color regulatory activities of specific inhibitors and/or activators to prove their validity [6,7]. The evaluation of skin color was thought to be particularly appropriate for phenotypic screens, as we can judge alterations visually and clearly. Although the availability of human skin tissues is certainly limited and the throughput utilizing them is insufficient, we are sure to obtain stable and reliable data that more than compensate for those disadvantages. However, we are often forced to give up on potential therapeutics because of concerns about their possible side effects when considering applications, even when candidate materials are found. In such cases, analysis of the mechanism involved is necessary so as not to waste the effort and expense invested.

Among the variety of methods used to uncover protein functions, chemical genetics is considered to be a promising approach, in which small molecule compounds are used as probes to elucidate protein functions within various signaling pathways [8,9]. While many kinds of methods and technologies have been developed to identify the target proteins of bioactive compounds to overcome one of the significant hurdles in chemical genetic research [10,11], the traditional approach using affinity chromatography has been successful in identifying the biological targets of multiple small molecules [12].

Several years ago, metformin, a drug widely used to treat type 2 diabetes, was reported to have antimelanogenic effects on reconstituted human epidermis and on human skin biopsies [13]. Since metformin belongs to the family of biguanide drugs, we were interested in the effects of other biguanide compounds on skin color regulation. Therefore, in the present study, we evaluated those using our ex vivo skin culture system and found that phenformin has a surprising skin tanning effect. Herein, we describe the drastic cutaneous darkening induced by phenformin and our elucidation of its mechanism of action using a forward chemical genetics approach, which led to the revelation of new promising bioactive materials.

## 2. Results

### 2.1. Phenformin Substantially Darkens Human Skin in a Dose-Dependent Manner

**Figure 1:**
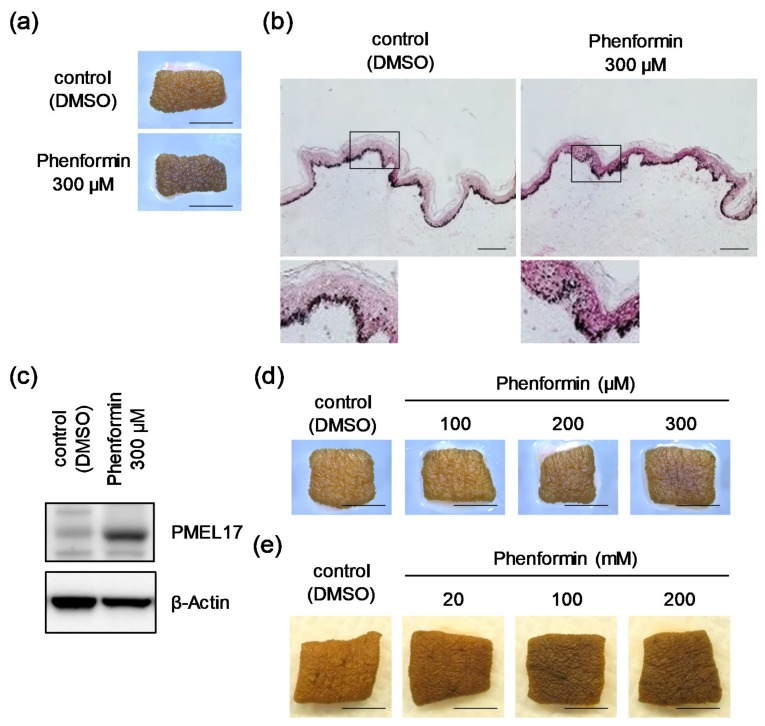
Skin samples from African-American (AA) donors are significantly darkened by phenformin in tissue culture. (**a**) Skin tissues obtained from an AA subject (25y) were subjected to tissue culture with or without 300 μM phenformin for 6 days. The photographs shown are representative samples. Scale bars = 5 mm. (**b**) Aforementioned skin tissues were then subjected to Fontana-Masson staining. Scale bars = 100 μm. The areas indicated by the squares are shown at higher magnification under each image. (**c**) PMEL17 protein was analyzed by Western blot using whole skin tissues shown in Figure 1a. β-Actin = loading control. (**d**) Pieces of skin tissue obtained from an AA subject (43y) were cultured with or without phenformin for 4 days at the indicated concentrations. The photographs shown are representative samples. Scale bars = 5 mm. (**e**) Pieces of skin tissue obtained from an AA subject (34y) were cultured with topical treatment by phenformin or Dimethyl sulfoxide (DMSO) for 8 days (at the indicated concentrations). Photographs show representative samples. Scale bars = 5 mm.

Our research group has continuously screened materials for the potential to regulate human skin color using an ex vivo skin culture system. In this study, we were interested in and focused on biguanides including metformin, according to a previous report that showed its depigmenting effect in intact cells, as well as in mice and humans [13]. In the course of this screening, we surprisingly found a remarkable skin tanning effect of phenformin, a biguanide hypoglycemic agent with actions and utilizations similar to those of metformin [14,15] and a known activator of AMP-activated protein kinase (AMPK) [16]. When human skin tissues obtained from African-American (AA) donors were treated with 300 μM phenformin for 6 days, the skin color was apparently and visibly darkened (Figure 1a), which was consistent with an increase in melanin content throughout the epidermis, especially in the basal layer demonstrated by Fontana-Masson staining (Figure 1b). Concomitantly, Western blot analysis showed an increased level of the melanosomal protein PMEL17 in the epidermis of darkened skin after the treatment with phenformin (Figure 1c). In addition to confirmation of this darkening effect on human skin tissues obtained from Hispanic and Caucasian donors (Figure S1), its darkening effect was found to be dose-dependent (Figure 1d). Furthermore, the topical application of phenformin only from the epidermal surface also darkened skin clearly (Figure 1e).

### 2.2. Phenformin Suppresses Melanosome Degradation in Epidermal Cells

**Figure 2:**
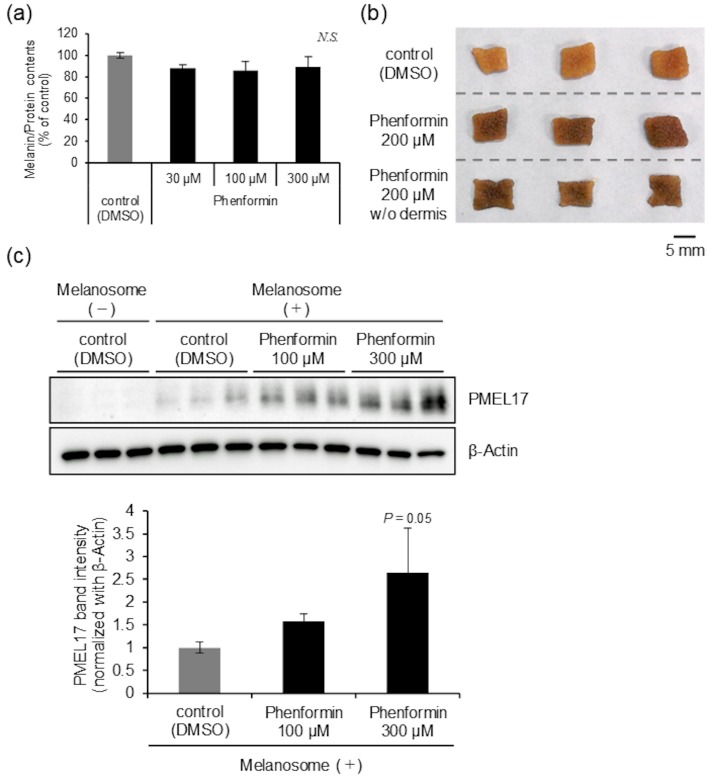
Phenformin decreases melanosome degradation in normal human epidermal keratinocytes (NHEKs). (**a**) Cellular melanin content of normal human epidermal melanocytes (NHEMs) treated with or without phenformin for 7 days (at the indicated concentrations). Values represent means ± SD of 3 individual samples. (**b**) Skin tissues with or without the dermis obtained from a Japanese subject (age unknown) were treated with or without 200 μM phenformin for 7 days. Photographs show representative samples. Scale bar = 5 mm. (**c**) NHEKs were cultured with isolated melanosomes for 24 h. After washing out unincorporated melanosomes, NHEKs were then cultured with or without phenformin for another 72 h (at the indicated concentrations). Amounts of PMEL17 protein were determined by Western blot. β-Actin = loading control. In addition, Western blots of PMEL17 and β-Actin proteins were analyzed quantitatively. Graph shows the relative expression level of PMEL17 normalized with β-Actin (band intensities). Values show means ± SD from 3 different samples treated with DMSO or phenformin after melanosome incorporation (*p* = 0.05 versus control (DMSO treatment) by Dunnett’s test).

As we have never seen such a change in skin color as induced by phenformin when other materials were screened using the same system, characterization of the underlying mechanism was quite fascinating. When we initially examined the effect of phenformin on melanin synthesis by normal human epidermal melanocytes (NHEMs), Dihydroxyphenylalanine (DOPA) oxidase activity, a critical rate-limiting step in melanogenesis [17], the expression levels of melanogenesis-related proteins, including Tyrosinase (TYR) and Tyrosinase-related protein 1 (TRP1) [18] and intracellular melanin content were not changed at a concentration less than 300 μM that did not affect the viability of NHEMs (Figure 2a, Figure S2). On the other hand, phenformin intensely darkened the skin regardless of whether the dermis was removed or not (Figure 2b). In addition, we observed a significant increase in the accumulation of incorporated melanosomes derived from MNT-1 cells in normal human epidermal keratinocytes (NHEKs) in a dose-dependent manner when NHEKs were treated with phenformin for 3 days (Figure 2c). These results led us to hypothesize that phenformin predominantly affects epidermal keratinocytes but not melanocytes and other types of dermal cells. Therefore, we subsequently aimed to characterize the cellular machinery involved in the cutaneous darkening, focusing on epidermal keratinocytes using a forward chemical genetics approach with phenformin as a bioprobe.

### 2.3. Identification of DHCR7 as a Phenformin-Binding Protein in Epidermal Cells

**Figure 3:**
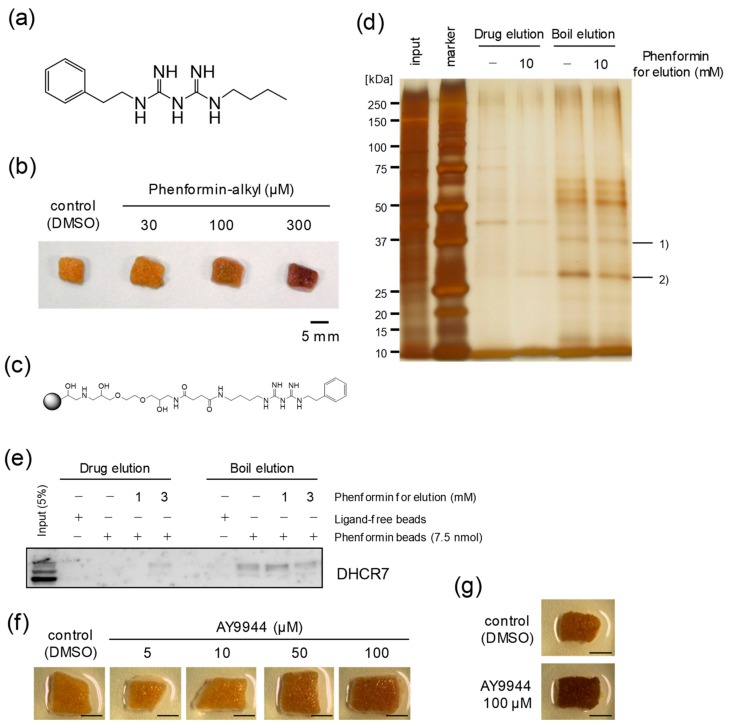
Identification of phenformin-binding proteins. (**a**) Chemical structure of phenformin-alkyl form. (**b**) Skin tissues obtained from a Japanese subject (age unknown) were treated with or without phenformin-alkyl form for 7 days (at the indicated concentrations). The photographs shown are representative samples. Scale bar = 5 mm. (**c**) Chemical structure of phenformin-immobilized FG-beads (phenformin-beads). (**d**) Lysates of NHEKs were incubated with phenformin-beads (7.5 nmol) for 4 h. After unattached beads were washed out, the co-precipitated proteins were eluted as described in the “Materials and Methods”. The co-precipitated proteins, bands marked 1) and 2), with phenformin-beads were identified by LC-MS/MS analysis. (**e**) Confirmation of the binding of phenformin to DHCR7 by Western blot analysis using an anti-DHCR7 antibody. (**f**) Skin tissues obtained from a Japanese subject (47y) were incubated with or without AY9944 for 7 days (at the indicated concentrations). The photographs shown are representative samples. Scale bars = 5 mm. (**g**) Skin tissues obtained from another Japanese subject (55y) were cultured with or without 100 μM AY9944 for 7 days. The photographs shown are representative samples. Scale bars = 5 mm.

In order to elucidate the mechanism underlying the cutaneous darkening caused by phenformin, we tried to identify the target protein(s) responsible for the suppression of melanosome degradation elicited by phenformin in NHEKs. Based on the structure-activity relationship (SAR) analysis of biguanide compounds regarding their skin tanning effects (Figure S3), we speculated that one or more amino groups of phenformin are not responsible for the tanning activity but that the phenyl group is probably involved (Figure S4). We then prepared FG-beads (commercialized magnetic beads) [19] to immobilize phenformin on their surface (Figure S5). After confirmation that the phenformin-alkyl form (Figure 3a), a reaction intermediate just before the immobilization on beads, was biologically active with the same potency as phenformin (Figure 3b), phenformin-immobilized FG-beads (phenformin-beads) were eventually synthesized (Figure 3c). Following incubation of lysates of NHEKs with phenformin-beads for 4 h, phenformin-beads were precipitated and then washed, after which co-precipitated proteins were eluted in two manners. First, phenformin-beads were dispersed in 10 mM phenformin (drug elution) or DMSO, and supernatants were collected after magnetic separation. Second, the remaining beads were boiled in SDS sample buffer (boil elution) and supernatants were collected again. Both sets of eluted proteins were separated by SDS-PAGE and detected by silver staining. In the stained gels, we observed two major protein bands that specifically co-precipitated with phenformin-beads, whose band signals in the boil eluted fraction got weaker through the process of drug elution with excess phenformin, and we identified those proteins by LC-MS/MS system as: (1) DHCR7 [20], and (2) SLC25A5 and/or SLC25A6 [21] (Figure 3d). Because SLC25A5 and/or SLC25A6 are classified in the mitochondrial carrier superfamily and are the most abundant proteins in the inner mitochondrial membrane [22], we suspected that they were the target protein(s). That would account for the lactic acidosis known as a lethal side-effect caused by the action of phenformin against the mitochondrial electron transport chain [23], which would explain the reason why phenformin disappeared from the market. On the other hand, since the binding of phenformin to DHCR7 was also confirmed by Western blotting of phenformin-bead-bound proteins using an anti-DHCR7 antibody (Figure 3e), we examined whether the functional suppression of DHCR7 could affect human skin color. Although our attempts to knockdown DHCR7 protein levels with RNA interference were not successful despite a reduction of its transcript level, we validated the involvement of DHCR7 in the cutaneous darkening induced by phenformin using AY9944, a specific DHCR7 inhibitor [24]. When human skin tissue obtained from a Japanese donor was treated with AY9944 for 7 days, the skin color was clearly darkened in a dose-dependent manner as expected (Figure 3f). The darkening effect of inhibiting DHCR7 was confirmed to be stably reproducible in an experiment with skin tissues obtained from another Japanese donor (Figure 3g).

### 2.4. Phenformin as well as AY9944 Suppresses Autophagic Activity, Leading to the Accumulation of Melanosomes in Epidermal Cells

**Figure 4:**
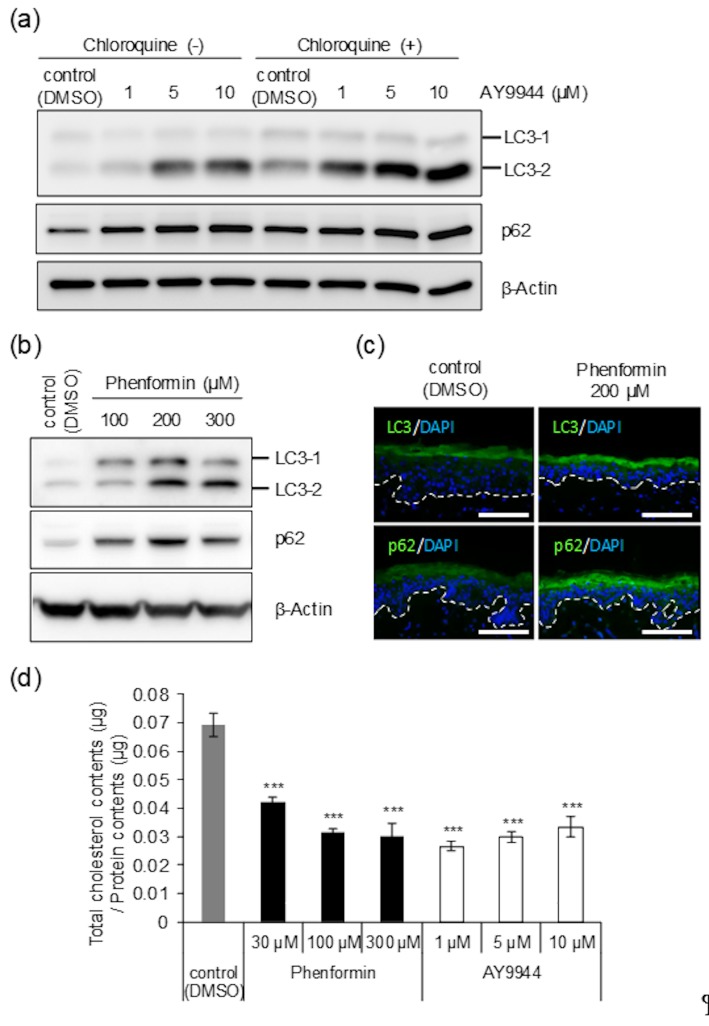
AY9944 diminishes autophagic activity in NHEKs similar to phenformin. (**a**) NHEKs were treated with or without AY9944 for 24 h (at the indicated concentrations) and were then further cultured with or without 10 μM chloroquine for another 24 h. After the incubation, proteins were harvested for Western blot analysis of LC3 and p62 proteins. β-Actin was used as a loading control. (**b**) Skin tissues obtained from an AA subject (43y) were treated with or without phenformin for 4 days (at the indicated concentrations). After the treatment, proteins were extracted for Western blot of LC3 and p62 proteins. β-Actin = loading control. (**c**) Skin tissues obtained from the AA subject above were treated with or without 200 μM phenformin and were used for immunofluorescence staining with an anti-LC3-antibody and an anti-p62 antibody. Scale bars = 100 μm. (**d**) NHEKs were treated with DMSO, phenformin or AY9944 for 3 days (at the indicated concentrations) and then collected for the quantification of cellular cholesterol content. Values are means ± SD of 3 independent samples (*** *p* < 0.001 versus control (DMSO treatment) by Dunnett’s test).

Based on the results reported above, we hypothesized that phenformin targets DHCR7 and suppresses its enzymatic activity in the final step of cholesterol biosynthesis according to a previous report [25], which would explain the inhibition of autophagosome formation due to the lack of cholesterol as one of its major components [26]. If this hypothesis was correct, it could easily fit in with our previous finding that autophagy plays a significant role in determining skin color by regulating melanosome degradation in NHEKs [6]. To address this, we performed autophagic flux assays in which the degradation of both LC3-Ⅱ (a marker of autophagosomes) and p62 (a known substrate of autophagy in autolysosomes) is assessed in the presence or absence of an autophagic inhibitor such as chloroquine, a lysosome inhibitor. Essentially, upon inhibition of autolysosome function, the reduction of autophagic activity should be reflected by the accumulation of LC3-Ⅱ and p62. If the expression levels of those proteins increase after the treatment with chloroquine, the occurrence of autophagic turnover can be inferred [27]. When NHEKs were treated with AY9944, LC3-Ⅱ and p62 were up-regulated in a dose-dependent manner. Given that the up-regulation was intensified in the presence of chloroquine, we examined whether AY9944 suppresses autophagic activity in NHEKs (Figure 4a). Additionally, the up-regulation of those proteins that reflects the suppression of autophagic activity was also confirmed in human skin tissues treated with phenformin and analyzed by Western blot (Figure 4b) and immunofluorescence (Figure 4c). Furthermore, the results demonstrated that phenformin as well as AY9944 suppressed cholesterol synthesis in NHEKs, which supports our hypothesis even though a dose-dependency of AY9944 was not observed, possibly due to its slight cell toxicity (Figure 4d).

### 2.5. Induction of Cutaneous Darkening by Berberine Emphasizes the Involvement of Cholesterol Biosynthesis in Skin Color Development

**Figure 5:**
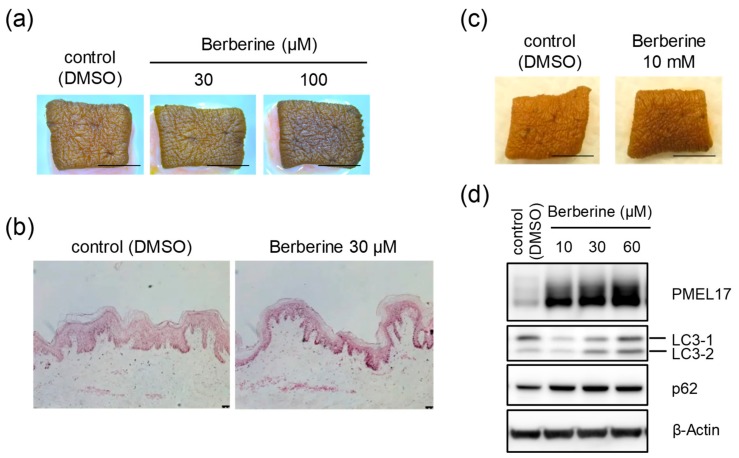
Berberine induces skin darkening through the inhibition of melanosome degradation in NHEKs. (**a**) Skin tissues obtained from an AA subject (34y) were subjected to tissue culture with or without berberine for 8 days (at the indicated concentrations). Photographs show representative samples. Scale bars = 5 mm. (**b**) Skins obtained from the AA subject above were treated with or without 30 μM berberine and then subjected to Fontana-Masson staining. Scale bars = 50 μm. (**c**) Skin tissues obtained from the AA subject above were also subjected to tissue culture by topical application with or without 10 mM berberine for 8 days. Photographs show representative samples. Scale bars = 5 mm. (**d**) NHEKs were cultured with isolated melanosomes for 24 h. They were subsequently washed, and then cultured with or without berberine for another 24 h (at the indicated concentrations). After the incubation, proteins were harvested for Western blot analysis of PMEL17, LC3 and p62 proteins. β-Actin = loading control.

To obtain positive proof for the relationship between cholesterol biosynthesis and skin color formation, we tried to verify this from a different perspective. If the provision of cholesterol is essential, materials other than AY9944 that inhibit enzymes in the cholesterol biosynthesis pathway should cause cutaneous darkening in a similar manner. Thus, we examined the effects of berberine, known as an inhibitor of 3-hydroxy-3-methylglutaryl coenzyme A (HMG-CoA) reductase, which catalyzes the rate-limiting step in cholesterol biosynthesis [28]. When human skin tissues obtained from AA donors were treated with berberine for 8 days, the skin color was visibly darkened (Figure 5a), which was also indicated by Fontana-Masson staining (Figure 5b). Additionally, the skin darkening effect was confirmed by the topical application of berberine (Figure 5c). Moreover, Western blot analysis demonstrated the suppression of melanosome degradation via the down-regulation of autophagic activity in NHEKs cultured with isolated melanosomes and treated with berberine (Figure 5d).

### 2.6. Berberine Affects Not only Melanosome Degradation in Epidermal Cells but also Melanin Synthesis in Melanocytes

**Figure 6:**
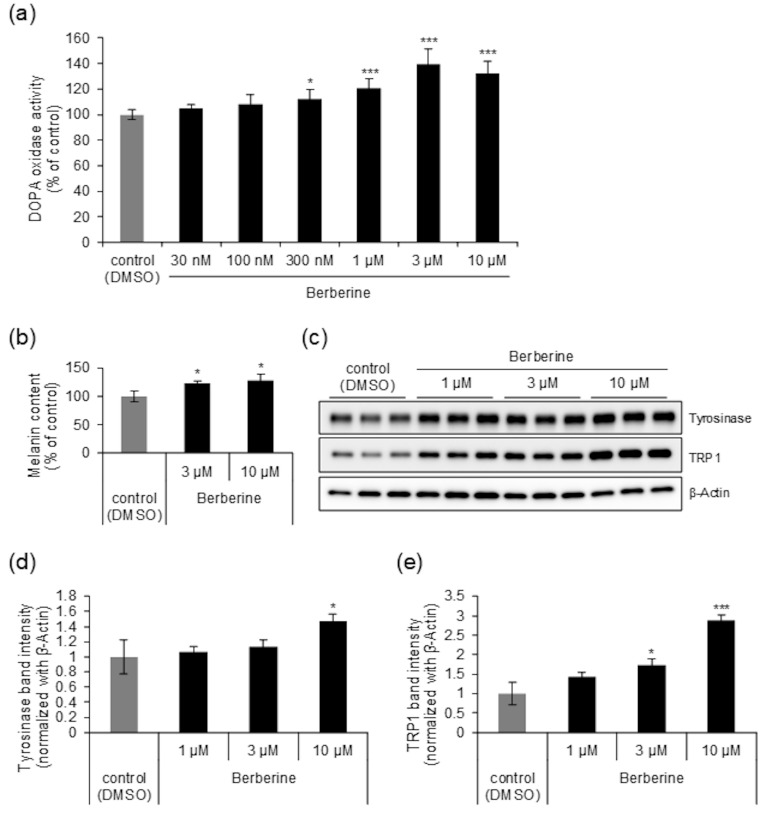
Berberine promotes melanin synthesis in NHEMs. (**a**) After incubation with or without berberine for 4 days (at the indicated concentrations), Dihydroxyphenylalanine (DOPA) oxidase activity in NHEMs was measured. Values represent means ± SD of 6 independent samples (*p < 0.05; ***p < 0.001 versus control (DMSO treatment) by Dunnett’s test). (**b**) NHEMs were treated with or without berberine for 6 days (at the indicated concentrations) and then collected for the quantification of cellular melanin content. Values represent means ± SD of 3 individual samples. (**c**) After incubation with or without berberine for 4 days (at the indicated concentrations), NHEMs were lysed and subjected to Western blot analysis of Tyrosinase and TRP1 proteins. β-Actin = internal control. (**d**,**e**) Quantitative analysis of the Western blots shown in Figure 6c. Values represent means ± SD of 3 separate samples treated with DMSO or berberine (* *p* < 0.05; *** *p* < 0.001 versus control (DMSO treatment) by Dunnett’s test).

Interestingly, in addition to its effects on NHEKs, we found that the effects of berberine on NHEMs were different from those of phenformin. After NHEMs were treated with berberine for 4 days, DOPA oxidase activity was significantly up-regulated in a dose-dependent manner (Figure 6a). In addition, a significant increase in intracellular melanin content was also consistently observed after treatment with berberine for 6 days (Figure 6b). In agreement with the increases in tyrosinase activity as well as melanin content elicited in NHEMs by berberine, the protein expression levels of TYR and TRP1 in NHEMs were also significantly up-regulated after the treatment with berberine for 4 days (Figure 6c–e). Thus, the skin tanning effect elicited by berberine is thought to result from the up-regulation of melanogenesis in epidermal melanocytes as well as the down-regulation of melanosome degradation in epidermal keratinocytes.

## 3. Discussion

In this study, we explored novel skin color regulators among biguanide compounds screened by our ex vivo skin culture system, which resulted in the unexpected and surprising finding that phenformin possesses a significant skin darkening capability despite our assumption that skin lightening compounds would be identified as well as metformin, a biguanide compound, whose effect on pigmentation was previously reported [13]. While the activation of AMPK and inhibition of the mitochondrial electron transfer system are known modes of action of biguanide compounds [29], the involvement of AMPK independent pathways has also been recently reported depending on physiological actions [30]. In fact, the depigmenting effect of metformin was suggested to possibly be attributed to AMPK independent pathways accompanied by reduced cAMP concentrations in melanocytes [13]. Likewise, while the phosphorylation of AMPK in NHEKs treated with phenformin was confirmed in our study (data not shown), phenformin-induced skin tanning was presumed to be independent of the AMPK pathway.

Thus, turning our attention to previous achievements in chemical genetics studies, we aimed to identify the target protein(s) of phenformin using FG-beads, high-performance affinity magnetic beads, which Handa and co-workers developed to overcome several disadvantages of conventional matrices, including the nonspecific binding of irrelevant proteins to the affinity matrices and the instability of those matrices [19]. Fortunately, we succeeded in identifying DHCR7 as a candidate target protein that binds phenformin, using our chemical pull-down assay with phenformin-immobilized FG-beads in lysates of NHEKs. Since similar cutaneous darkening was also induced when an ex vivo skin culture system was treated with AY9944, DHCR7 was conclusively suggested to be the target of phenformin in our study. In addition, subsequent investigation led us to hypothesize that phenformin induces the accumulation of epidermal melanin by inhibiting autophagic activity through the suppression of cholesterol biosynthesis in epidermal keratinocytes. Apart from the inhibition of DHCR7 by a specific inhibitor (AY9944), we also focused on HMG-CoA reductase, another key enzyme in the cholesterol biosynthesis pathway, as an alternative approach to verify the essential role of cholesterol biosynthesis as well as cholesterol homeostasis in skin color development because it has been found that the rates of forward and reverse cholesterol transport and cholesterol synthesis are under tight control in order to maintain cellular cholesterol balance and that sterol regulatory element-binding protein 2 is activated by low cellular cholesterol and acts to increase cellular cholesterol levels by facilitating synthesis and uptake and decreasing efflux [31]. In addition, reduced cellular cholesterol levels by blocking cholesterol synthesis have been reported to lead to an up-regulation of cholesterol receptors such as a low-density lipoprotein receptor and scavenger receptor class B type 1 (SR-BI), a high-density lipoprotein receptor [32,33]. Furthermore, a recent paper interestingly reported a strong correlation of the expression of SR-BI with MITF expression as well as with the melanin synthesis pathway in human melanoma cells [34]. Since not only AY9944, but also berberine, an inhibitor of HMG-CoA reductase, had consistent potency for skin darkening accompanied by a diminished autophagic activity, it is reasonable to think that there is a close relationship among cholesterol synthesis and/or homeostasis, autophagic activity and skin color regulation. However, it cannot be concluded yet how statin-based drugs with an inhibitory activity on HMG-CoA reductase [35] affect melanogenic activity in melanocytes because of several inconsistent results [36,37,38,39], whereas berberine dose-dependent increases in the expression of melanogenesis-related proteins, tyrosinase activity and melanin content in NHEMs were consistently observed in this study. We now suggest for the first time that cholesterol synthesis and/or homeostasis, at least in epidermal keratinocytes, substantially contributes to the regulation of skin color.

Our motivation to identify materials that effectively control skin color is really attributed to our enthusiasm to help patients who suffer from skin pigmentation disorders such as vitiligo. Phenformin, which was once approved by the Food and Drug Administration as an anti-diabetic drug, has been banned from use in most countries since severe lactic acidosis has become apparent as an adverse drug reaction, although the same symptoms may not be induced by topical application. Thus, we needed to switch our strategy to search for alternative materials with comparable effects based on the mechanism involved. From this viewpoint, the new finding of the skin darkening effect of berberine is valuable new knowledge obtained in this research. Berberine, an isoquinoline alkaloid isolated from multiple herbs such as *Coptis chinensis Franch*, is a current over-the-counter (OTC) drug in China for treating microbial diarrhea [40]. Berberine is also currently sold in the US as a dietary supplement. In addition, the Goldenseal extract derived from *Hydrastis canadensis* that contains berberine appears to be manufactured for external use such as in cosmetics. Interestingly, Barberry root, which contains berberine in abundance, can be used for the treatment of vitiligo, due to its anti-inflammatory and immunomodulatory properties that are expected to protect melanocytes by suppressing their autoimmune destruction, to stop the depigmentation and to start the re-pigmentation of vitiligo patches [41]. Moreover, we recently found an intriguing study reporting that berberine darkens the skin of *Bufo melanostictus* caused by a mechanism whereby berberine stimulates β2-adrenergic receptors to alter the tissue distribution of melanophores [42]. In addition to those findings, we demonstrated that berberine has dual effects, not only suppressing melanosome degradation in keratinocytes but also promoting melanin synthesis in melanocytes. Therefore, it would be reasonable to develop effective skin darkening agents by optimizing berberine with a serious effort. In the future, we will continue this research to accumulate knowledge with the aim of technical and/or material developments for medical use.

In summary, we show for the first time a realistic methodology to explore compounds that can regulate skin color using a skin tissue culture system. Our methodology allowed us to identify phenformin, which had a surprising skin darkening activity and to analyze its underlying mechanism of action. Although further studies are needed, our results demonstrate that cholesterol biosynthesis in epidermal keratinocytes contributes to the regulation of skin color. Our study also identified berberine, which is a material worthy of consideration for vitiligo treatment in the future due to its substantial skin tanning effect. Our findings not only provide new insights for the fundamental understanding of the mechanisms that underly cutaneous pigmentation homeostasis but also suggest new strategies to explore materials for both cosmetic and therapeutic applications towards skin color refinement.

## 4. Materials and Methods

### 4.1. Materials

Normal human epidermal melanocytes (NHEMs; donor skin type: dark) were obtained from Kurabo (Osaka, Japan) and normal human epidermal keratinocytes (NHEKs; donor skin type: light) were obtained from Thermo Fisher Scientific (Waltham, MA, USA). MNT-1 melanoma cells were kindly provided by Dr. Pier Giorgio Natali (Regina Elena Institute, Rome, Italy). The anti-β-actin-specific antibody was supplied by Sigma-Aldrich (St. Louis, MO, USA). The human PMEL17-specific antibody (clone HMB-45) was acquired from DAKO (Carpinteria, CA, USA). Antibodies against other human proteins were acquired from the following sources: anti-DHCR7 (Abcam, Cambridge, UK); anti-Tyrosinase and anti-LC3 (Life Technologies, Carlsbad, CA, USA); anti-TRP1 (Santa Cruz Biotechnology, Dallas, TX, USA); anti-p62 (MBL, Aichi, Japan). Phenformin hydrochloride (phenformin), 1-phenylbiguanide hydrochloride (phenylbiguanide) and metformin hydrochloride (metformin) were obtained from Sigma Aldrich. Buformin hydrochloride (buformin) was purchased from Wako Pure Chemical Industries (Osaka, Japan). N-[amino(imino)methyl]piperidine-1-carboximidamide was purchased from Matrix Scientific (Columbia, SC, USA). AY9944 dihydrochloride (AY9944) was obtained from Abcam. Berberine hydrochloride (berberine) was obtained from Nacalai tesque, Inc (Kyoto, Japan). FG-beads were purchased from Tamagawa Seiki Co., Ltd. (Nagano, Japan). Other chemicals were of reagent grade.

### 4.2. Human Skin

Surgically excised adult female skin tissues obtained from Caucasian, African American and Hispanic donors were provided by the National Disease Research Interchange (NDRI, Philadelphia, PA, USA). The collection of those skin tissues was approved by the Institutional Review Board of IntegReview Ltd. (Austin, TX, USA). Surgically excised adult female skin tissues from Japanese donors were provided by Jichi Medical University (Tochigi, Japan). The collection of those skin tissues was approved by the Kao Institutional Human Research Ethics Committee (Tokyo, Japan). This study was conducted according to the Declaration of Helsinki protocols and informed consent was obtained from each volunteer prior to the procedure.

### 4.3. Cell Culture

NHEKs were cultured in Epilife medium (Thermo Fisher Scientific) supplemented with 10 µg/mL insulin, 0.1 µg/mL human recombinant epidermal growth factor (EGF), 0.5 µg/mL hydrocortisone, 50 µg/mL gentamycin, 50 ng/mL amphotericin B and 0.4% (v/v) bovine pituitary extract (BPE) at 37 °C in an atmosphere of 5% (v/v) CO_2_. NHEMs were maintained in Medium 254 (Thermo Fisher Scientific) supplemented with 5 µg/mL insulin, 5 µg/mL transferrin, 3 ng/mL human recombinant fibroblast growth factor, 0.18 µg/mL hydrocortisone, 3 µg/mL heparin, 10 ng/mL phorbol 12-myristate 13-acetate, 0.2% (v/v) BPE and 0.5% (v/v) fetal bovine serum (FBS) at 37 °C in an atmosphere of 5% (v/v) CO_2_. MNT-1 cells were preincubated in RPMI-1640 medium supplemented with 10% (v/v) FBS and 10% (v/v) AIM-V medium (Life Technologies). NHEKs were seeded in 6-well plates (Becton Dickinson Labware, Franklin Lakes, NJ, USA) at a density of 1.0 × 10^5^ cells per well and then incubated for 24 h, followed by incubation in medium without BPE and human recombinant EGF. Following incubation for another 24 h, cells were treated with the indicated reagents or melanosomes isolated from MNT-1 melanoma cells [43], and were subsequently incubated according to the indicated experimental design.

### 4.4. Measurement of DOPA Oxidase Activity

DOPA oxidase activity was measured as described elsewhere [17]. Briefly, 1.0 × 10^4^ NHEMs in 96-well plates were washed twice with phosphate-buffered saline (PBS) and then mixed with 20 μL Buffer A (0.1 M Tris-HCl, pH 7.2, 1% NP-40 and 0.01% SDS) and with 20 μL Buffer B (100 mM sodium phosphate, pH 7.1) and 4% N, N’-dimethylformamide (DMF). After incubation at 4 °C for 2 h, 80 μL Buffer B, 60 μL 20.7 mM 3-methyl-2-benzothiazolinonehydrazone hydrochloride and 40 μL L-DOPA were added to each well. After reaction for 30 min at 37 °C, the absorbance of each well at 505 nm was measured using a Gen 5 Micro Plate Reader (BioTek Instruments, Winoski, VT, USA).

### 4.5. Measurement of Melanin Content in NHEMs

After incubation with compounds at the indicated concentrations, NHEMs were washed with PBS and collected in 1.5 mL tubes using a cell scraper. After centrifugation at 15,000 rpm for 10 min, the cell pellets were solubilized in 150 μL 2 M NaOH and melanin contents were measured using a Gen 5 Micro Plate Reader (BioTek Instruments) at 405 nm.

### 4.6. Melanosome Incorporation into NHEKs

NHEKs were incubated with melanosomes isolated from MNT-1 cells as described above. Twenty-four hours after that treatment, non-incorporated melanosomes were removed by washing three times with PBS, followed by subsequent incubation for an additional 24 h. Cells were then collected for Western blot analysis as described below.

### 4.7. Western Blot Analysis

Samples (cells or human skin tissues) were washed with PBS, solubilized in 0.1 mL RIPA buffer (Sigma-Aldrich) supplemented with 1 mM phenylmethylsulfonyl fluoride (PMSF) (Sigma-Aldrich) and then homogenized using ultrasonication. The protein concentrations of the resulting supernatants were determined using the BCA protein assay reagent (Pierce Biotechnology, Rockford, IL, USA). The whole cell-lysates were separated using 10, 12.5 and 4–15% SDS-polyacrylamide gels (Bio-Rad Laboratories) and were then transferred to Sequi-Blot polyvinylidene difluoride membranes (Bio-Rad Laboratories). Those membranes were incubated with primary antibodies specific for the indicated proteins followed by incubation with appropriate secondary antibodies (anti-mouse IgG peroxidase-linked F[ab]^2^ fragment (GE Healthcare, Buckinghamshire, UK) or anti-rabbit IgG peroxidase-linked F[ab]^2^ fragment (GE Healthcare). Subsequent visualization of the bound antibodies was performed using Enhanced ChemiLuminescence Prime (GE Healthcare) with a LAS4000 imaging system (Fujifilm, Tokyo, Japan) or an ODYSSEY Fc imaging system (LI-COR Inc., Lincoln, NE, USA) according to the manufacturer’s instructions. Densities of the detected bands were analyzed using Image J software.

### 4.8. Preparation of Phenformin-Immobilized FG-Beads

Phenylethylamine hydrochloride (1 g, 6.3 mmol, 1 eq) and sodium dicyanamide (678 mg, 7.6 mmol, 1.2 eq) were added to 15 mL 2-propanol and the suspension was stirred at 90 °C overnight. The reaction mixture was filtered and the filtrate was evaporated to afford 1-cyano-3-phenethyl guanidine as a white solid.

N-(t-butoxycarbonyl)-1,4-diaminobutane hydrochloride (133 mg, 0.6 mmol, 1 eq) and 1-cyano-3-phenethyl guanidine (134 mg, 0.7 mmol, 1.2 eq) were suspended in 4 mL BuOH and stirred overnight at 100 °C under an argon atmosphere. The reaction mixture was evaporated and the residue was purified by flash column chromatography (SiO_2_, chloroform/methanol = 9/1) to afford N^1^-{N-(t-butoxycarbonyl)-aminobutyl}-N^5^-phenetylbiguanidine (18.1 mg, 8%).

N^1^-{N-(t-butoxycarbonyl)-aminobutyl}-N^5^-phenetylbiguanidine (2.1 mg, 5.6 μmol, 1 eq) was dissolved in 500 μL MeOH and mixed with 500 μL 4N HCl /1,4-dioxane and then stirred for 20 min. The solution was evaporated to give N^1^-aminobutyl-N^5^-phenetylbiguanidine hydrochloride as the phenformin probe.

Magnetic FG beads (NHS beads; TAS8848 N1141, 2.5 mg) were incubated with the phenformin probe at low (0.1 mM) and at high (0.3 mM) concentrations in the presence of triethylamine at 0.2 mM and 0.6 mM, respectively, in 500 μL DMF at 20 °C for 90 min. After removing the DMF, unreacted amino groups on the FG beads were masked using 1 M aminoethanol in 500 μL DMF for 2 h and then washed with 50% MeOH and used for the affinity pull-down assays.

### 4.9. Identification of Phenformin-Binding Proteins (Chemical Pull-down Assay)

NHEKs were collected and sonicated four times for 10 s each in 100 mM KCl buffer (2 × 100 mM buffer [40 mL 2.5 M KCl buffer, 126 g glycerol, 20 mL 1 M HEPES (pH 7.5), 1 mL 1 M MgCl_2_, 200 μL 1 M CaCl_2_, 400 μL 0.5 M EDTA (pH 8.0), 1 mL Igepal CA-630 and double distilled water (DDW) to a total volume of 500 mL] diluted twice with DDW. The cell lysates were centrifuged at 15,000 rpm for 30 min at 4 °C. The resulting supernatants were precleared with FG-beads for 1 h and equal amount (0.8 mg) of protein lysates per sample was incubated with phenformin-immobilized beads or ligand-free beads for 4 h at 4 °C. The beads were washed three times with 100 mM KCL buffer. The bound proteins were eluted in 2 ways (First: elution with 1% (v/v) DMSO or phenformin/100 mM KCl buffer; Second: boiling in SDS sample buffer). The resulting solubilized proteins were subjected to SDS-PAGE followed by silver staining or immunoblotting. Following silver staining, bands corresponding to the phenformin binding proteins were excised, and the gel pieces were sent to Shimadzu Techno-Research, Inc. (Kyoto, Japan) and analyzed using an LC-MS/MS system to identify their constituent proteins.

### 4.10. Human Skin Culture

Human skin tissues were cut into about 1 cm^2^ pieces after trimming the subcutaneous fat tissue and were cultured in Advanced Dulbecco’s modified eagle medium (Thermo Fisher Scientific) supplemented with 10% (v/v) FBS at 37 °C in an atmosphere of 5% (v/v) CO_2_. When skin tissues were treated with various compounds, they were maintained in 6-well plates with the medium noted above with exchanges every two or three days. After completion of the cultures, images of them were obtained using a Leica S8 APO stereo microscope (Leica Microsystems, Bannockburn, IL) or a handy digital camera.

### 4.11. Fontana-Masson Staining

Skin samples after tissue culture were fixed with 10% buffered formalin and then embedded in paraffin. Melanin pigment was visualized using Fontana-Masson staining with an eosin counterstain as described previously [44].

### 4.12. Immunofluorescence Microscopic Analysis

Tissues were fixed on slides with acetone and were then permeabilized with 0.1 μg/mL Triton X-100 in PBS. Tissues were incubated in 2.5% normal horse serum (Vector, Burlingame, CA, USA), followed by treatment with a rabbit anti-LC3 antibody (Life Technologies; 1:500 dilution) or a rabbit anti-p62 antibody (MBL; 1:500 dilution). Tissues were then incubated with Alexa Fluor 488^®^ goat anti-rabbit IgG (H+L) highly cross-absorbed (Life Technologies; 1:1000 dilution), followed by staining of nuclei with 4’6-diamidino-2-phenylindole (DAPI) (Life Technologies; 1:2000 dilution). Slides were mounted in Fluoromount-G^®^ (Southern Biotech, Birmingham, AL, USA) and images were obtained using a Leica DM5500B digital microscope (Leica Microsystems).

### 4.13. Measurement of Intracellular Cholesterol Contents

NHEKs were seeded in 6-well plates at a density of 2.0 × 10^5^ cells per well and then incubated for 24 h, followed by incubation in medium without BPE and human recombinant EGF. After treatment with the indicated reagents for 3 days, NHEKs were washed with PBS and half of them (samples in 3 wells) were subjected to the measurement of intracellular cholesterol content using a Cholesterol/Cholesteryl Ester Quantitation Assay Kit (Abcam) according to the manufacturer’s instructions. The other half of the samples (samples in the other 3 wells) were solubilized in 0.1 mL RIPA buffer (Sigma-Aldrich) supplemented with 1 mM PMSF (Sigma-Aldrich), and then homogenized using ultrasonication for the determination of protein concentrations. Total cholesterol contents are shown after normalization with the protein content per well.

### 4.14. Statistics

Significance of differences was calculated by analysis of variance. A *p*-value < 0.05 is considered statistically significant.

## Supplementary Materials

Supplementary materials can be found at https://www.mdpi.com/1422-0067/21/4/1451/s1. Figure S1: Phenformin darkens skin samples from a Hispanic donor and a Caucasian donor in tissue culture. Figure S2: Phenformin does not affect DOPA oxidase activity and the protein expression levels of melanogenesis-related proteins in melanocytes. Figure S3: Evaluation of the potential effects of biguanide compounds on the regulation of skin color. Figure S4: SAR (structure-activity relationship) analysis of biguanide compounds to evaluate their potential skin darkening effects. Figure S5: Preparation of phenformin-immobilized FG-beads.

## Abbreviations

AA	African-American
AMPK	AMP-activated protein kinase
BPE	Bovine pituitary extract
DAPI	4’6-diamidino-2-phenylindole
DDW	Double distilled water
DHCR7	7-dehydrocholesterol reductase
DMF	N, N’-dimethylformamide
DMSO	Dimethyl sulfoxide
DOPA	Dihydroxyphenylalanine
EGF	Epidermal growth factor
FBS	Fetal bovine serum
HMG-CoA	3-hydroxy-3-methylglutaryl coenzyme A
NHEK	Normal human epidermal keratinocyte
NHEM	Normal human epidermal melanocyte
OTC	Over-the-counter
PBS	Phosphate-buffered saline
PMSF	Phenylmethylsulfonyl fluoride
SAR	Structure-activity relationship
SR-BI	Scavenger receptor class B type 1
TRP1	Tyrosinase-related protein 1
TYR	Tyrosinase
