# Supplementary material for: The Surprising Effect of Phenformin on Cutaneous Darkening and Characterization of Its Underlying Mechanism by a Forward Chemical Genetics Approach
Source: Int J Mol Sci. 2020 Feb 20;21(4):1451. doi: 10.3390/ijms21041451 (PMC7073119; doi:10.3390/ijms21041451)

Supplementary materials

Title

The surprising effect of phenformin on cutaneous darkening and characterization of its underlying mechanism by a forward chemical genetics approach

Journal

International Journal of Molecular Sciences

Authors

Kei Takano, Akira Hachiya, Daiki Murase, Akiko Kawasaki, Hirokazu Uda, Shinya Kasamatsu, Yoshiya Sugai, Yoshito Takahashi, Tadashi Hase, Atsushi Ohuchi and Tamio Suzuki

***Corresponding author:** Dr. Akira Hachiya, Planning and Implementation, Kao Corporation, Haga, Tochigi, 321-3497, JAPAN

Phone: +1-513-455-5532. E-mail: hachiya.akira@kao.com

Supplemental Figure Legends

Supplemental Figure 1. Phenformin darkens skin samples from a Hispanic donor and a Caucasian donor in tissue culture.

(a) Skin tissues obtained from a Hispanic subject (48y) were subjected to tissue culture with or without 300 μM phenformin for 8 days. The photographs shown are representative samples. Scale bars = 5 mm. (b) Treated skins were subjected to Fontana-Masson staining. Scale bars = 100 μm. The areas indicated by the squares are shown at higher magnification under each image. (c) Skin tissues obtained from a Caucasian subject (18y) were subjected to tissue culture with or without 300 μM phenformin for 6 days. The photographs shown are representative samples. Scale bars = 5 mm.

Supplemental Figure 2. Phenformin does not affect DOPA oxidase activity and the expression levels of melanogenesis-related proteins in melanocytes.

(a) After treatment of NHEMs with or without phenformin for 3 days (at the indicated concentrations), DOPA oxidase activity was measured. Values represent means ± SD of 6 independent samples (***P < 0.001 versus control (DMSO treatment) by Dunnett's test). (b) Microscopic images of NHEMs treated with DMSO (control) or phenformin (300 μM or 1 mM). (c) After incubation with or without phenformin for 4 days (at the indicated concentrations), NHEMs were lysed and subjected to western blot analysis of Tyrosinase and TRP1 proteins. β-Actin = internal control.

Supplemental Figure 3. Evaluation of the potential effects of biguanide compounds on the regulation of skin color.

Skin tissues obtained from a Japanese subject (age unknown) were subjected to tissue culture with

or without (a) phenformin, (b) phenylbiguanide, (c) metformin, (d) buformin or (e) N-[amino(imino)methyl]piperidine-1-carboximidamide for 7 days. The photographs shown are representative samples. Scale bars = 5 mm.

Supplemental Figure 4. SAR (structure-activity relationship) analysis of biguanide compounds to evaluate their potential skin darkening effects.

Chemical structures of (a) a skin darkening compound (phenformin), and (b) non-skin darkening compounds (phenylbiguanide, metformin, buformin and N-[amino(imino)methyl]- piperidine-1-carboximidamide) are shown. We speculate that amino groups of phenformin are not involved in the darkening activity in contrast to the phenyl group.

Supplemental Figure 5. Preparation of phenformin-immobilized FG-beads.

The reaction scheme shows the immobilization of phenformin on magnetic FG-beads. The experimental procedure is described in “Materials and Methods”.

↑

Supplemental Figure 1

Takano *et al*.

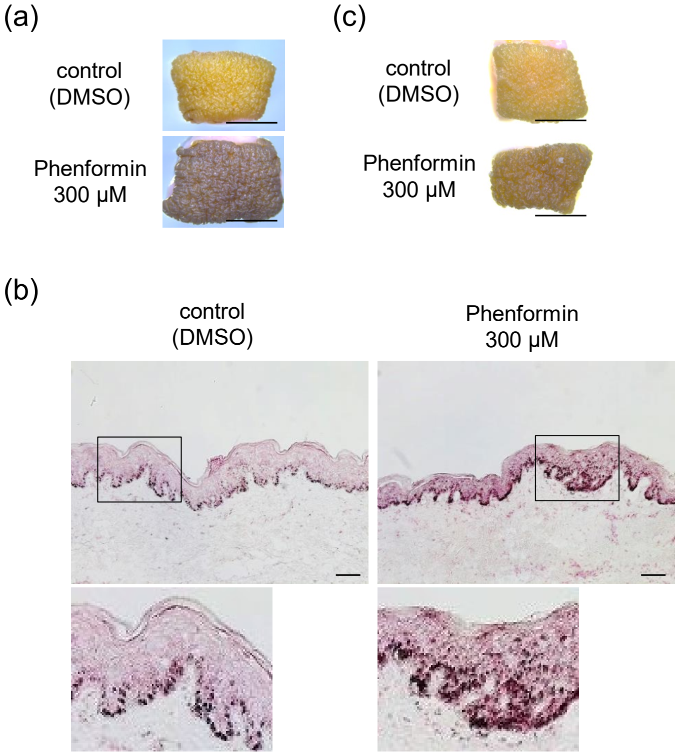

↑

Supplemental Figure 2

Takano *et al*.

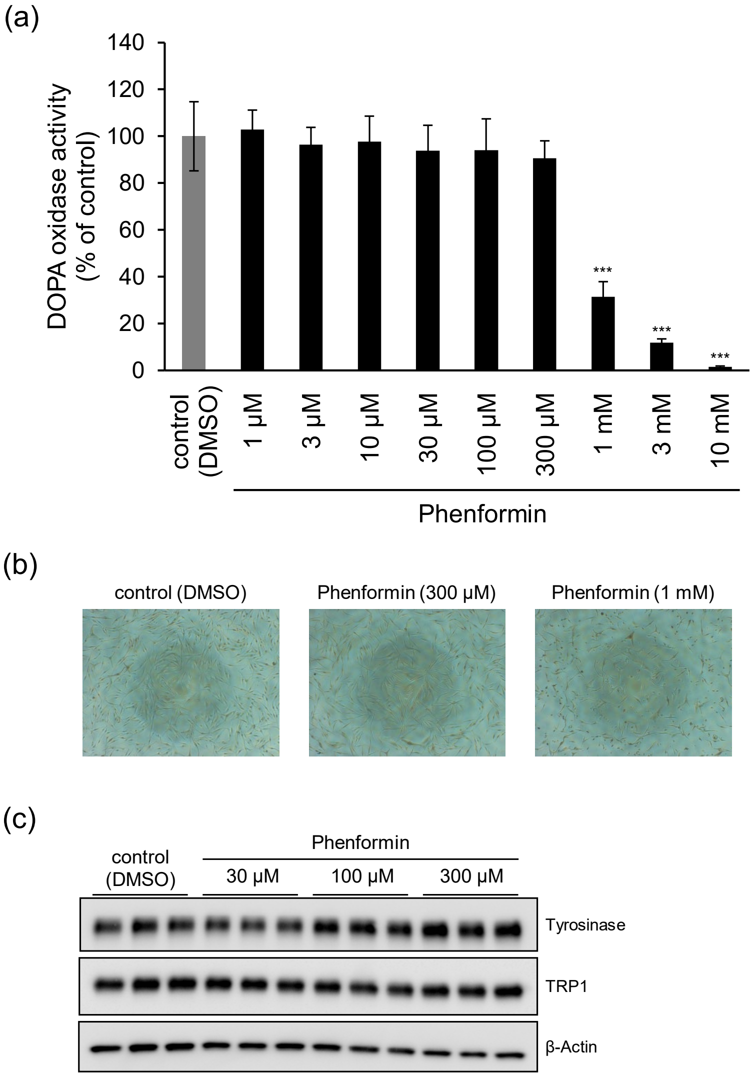

↑

Supplemental Figure 3
Takano *et al*.

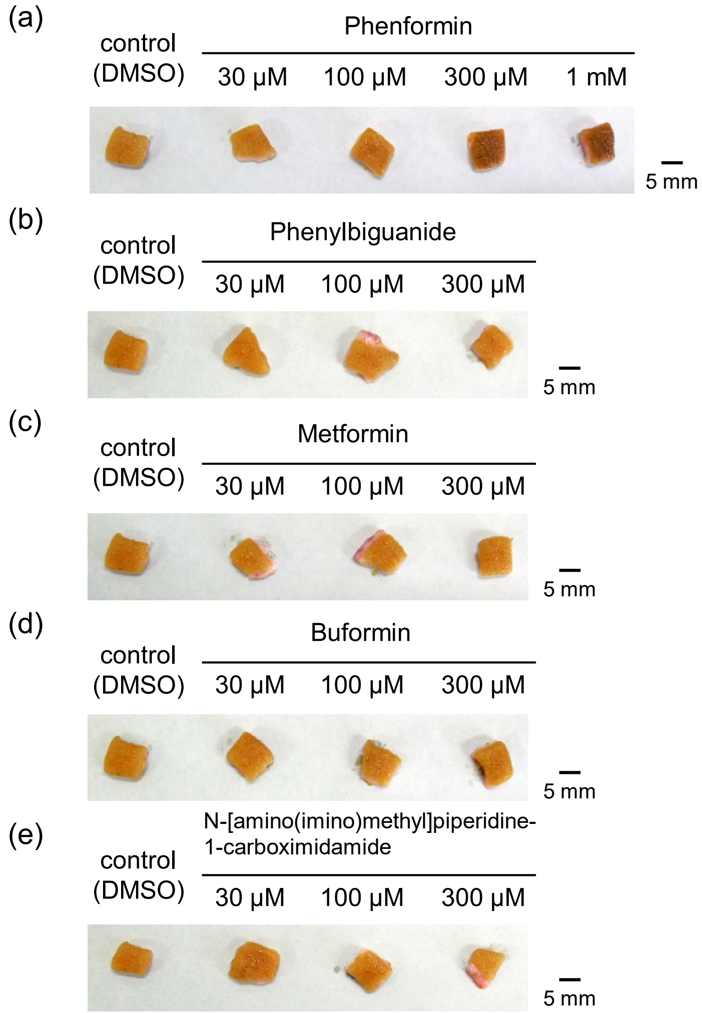

↑

Supplemental Figure 4

Takano *et al*.

(a) Skin darkening compound

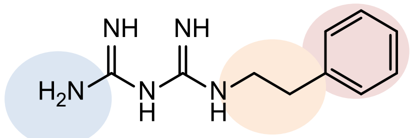

Phenformin

(b) non-skin darkening compounds

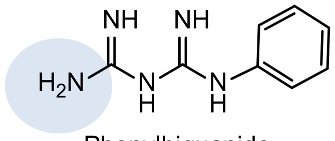

Phenylbiguanide

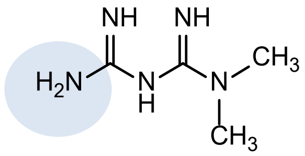

Metformin

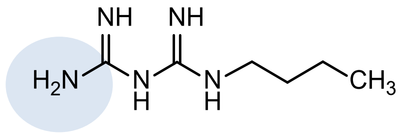

Buformin

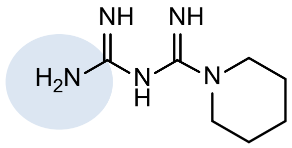

N-[amino(imino)methyl]piperidine-
1-carboximidamide

↑

Supplemental Figure 5

Takano *et al*.

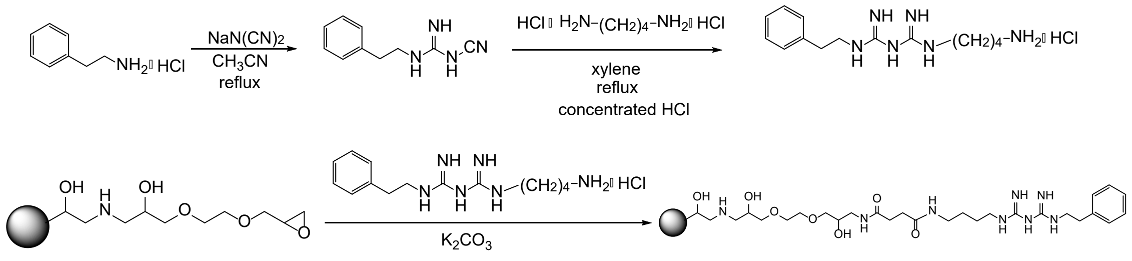

Supplement: Supplementary file 1 [file ijms-21-01451-s001.pdf]
